# The relation of airway obstruction to asthma, chronic rhinosinusitis and age: results from a population survey of adults

**DOI:** 10.1111/all.12447

**Published:** 2014-07-19

**Authors:** D Obaseki, J Potts, G Joos, J Baelum, T Haahtela, M Ahlström, P Matricardi, U Kramer, M Gjomarkaj, W Fokkens, J Makowska, A Todo-Bom, K Toren, C Janson, S-E Dahlen, B Forsberg, D Jarvis, P Howarth, G Brozek, J Minov, C Bachert, P Burney, for the GALEN network of excellence

**Affiliations:** 1 Department of Medicine, Obafemi Awolowo UniversityIle-Ife, Nigeria; 2 Respiratory Epidemiology and Public Health Group, National Heart and Lung Institute, Imperial College LondonLondon, UK; 3 Department of Respiratory Medicine, Ghent University HospitalGhent, Belgium; 4 Odense University Hospital, Odense UniversityOdense, Denmark; 5 Skin and Allergy Hospital, Helsinki UniversityHelsinki, Finland; 6 Department of Pediatric Pneumonology and Immunology, Charité-Universitätsmedizin BerlinBerlin, Germany; 7 IUF – Leibniz Research Institute for Environmental MedicineDüsseldorf, Germany; 8 Department of Dermatology and Allergy am Biederstein, Technical University MunichMunich, Germany; 9 Institute of Biomedicine and Molecular Immunology, National Research CouncilPalermo, Italy; 10 Department of Otorhinolaryngology, Academic Medical CenterAmsterdam, the Netherlands; 11 Department of Immunology, Rheumatology and Allergy, Medical University of LodzLodz, Poland; 12 Faculty of Medicine, University of CoimbraCoimbra, Portugal; 13 Section of Occupational and Environmental Medicine, University of GothenburgGothenburg, Sweden; 14 Department of Medical Sciences: Respiratory Medicine and Allergology, University of UppsalaUppsala, Sweden; 15 CfA - The Centre for Allergy Research, Karolinska InstituteStockholm, Sweden; 16 Occupational and Environmental Medicine, Umeå UniversityUmeå, Sweden; 17 Clinical and Experimental Sciences, Faculty of Medicine, Southampton General HospitalSouthampton, UK; 18 Department of Epidemiology, Medical University of Silesia in KatowiceKatowice, Poland; 19 Institute for Occupational Health of Republic of MacedoniaSkopje, Republic of Macedonia; 20 Upper Airway Research Laboratory, University of GhentGhent, Belgium; 21 Division of Ear, Nose, and Throat Diseases, Clintec, Karolinska InstituteStockholm, Sweden

**Keywords:** asthma, atopy, pulmonary function, rhinitis

## Abstract

**Rationale:**

There is conflicting evidence on whether patients with asthma experience an accelerated decline in lung function with age. We examined the association between postbronchodilator lung function, asthma, chronic rhinosinusitis (CRS), and atopy with age using a large European sample.

**Methods:**

In 17 centers in 11 European countries, case–control studies were nested within representative cross-sectional surveys of adults aged less than 75 years. Representative samples of participants with asthma, CRS or both and controls were assessed for postbronchodilator ventilatory function, smoking history, atopy, and treatment. Multiple regression was used to assess the interactive effects of age and diagnostic group on decline in postbronchodilator ventilatory function.

**Results:**

A total of 3337 participants provided adequate data (778 with asthma, 399 with CRS, 244 with both asthma and CRS and 1916 controls who had neither asthma nor CRS). Participants with asthma had lower FEV_1_/FVC (−4.09% (95% CI: −5.02, −3.15, *P* < 0.001) and a steeper slope of FEV_1_/FVC against age (−0.14%/annum [95%CI: −0.19, −0.08]) equivalent to smoking 1–2 packs of cigarettes per day. Those with atopy had a slope equivalent to controls.

**Conclusions:**

People with asthma have a steeper decline in postbronchodilator lung function with age, but neither CRS nor atopy alone were associated with such decline.

In 1987, Burrows and colleagues showed that subjects with diagnosed asthma and atopy but under ten pack years of cigarette smoking had both a slow decline in lung function and low mortality, whereas those without asthma or atopy but greater than 10 pack-years of smoking had both a rapid decline in lung function and a high mortality (1).

Consistent with these findings the Copenhagen City Heart Study reported that self-reported asthma was only associated with an increased rate of decline in prebronchodilator forced expiratory volume in one-second (FEV_1_) among those who developed asthma during the period of observation (2). The study subsequently confirmed Burrows' observation that the decline was independent of atopy (3). Among children it has been shown that growth of lung function is not affected by the presence of asthma, even though lung function is lower in children with asthma (4, 5).

Nevertheless both population (6–9) and clinical studies(10, 11) have provided evidence that a sub-group of asthmatics will develop fixed airway obstruction. These observations are supported by a community-based cross-sectional survey of adults in New Zealand which showed that the age-adjusted odds ratio for a postbronchodilator forced expiratory volume in one-second/forced vital capacity ratio (FEV_1_/FVC) <0.7 was 1.3 (95%CI: 1.1, 1.5) for ten pack years of smoking, 1.8 (95%CI: 1.1, 3.0) for atopy and 5.2 (95%CI: 2.5, 10.6) for a childhood history of asthma (12).

The evidence remains confusing because clinical impressions can be unduly influenced by biases in who returns to the clinic, and studies that rely on prebronchodilator lung function may have additional errors in assessing changes over time.

Accelerated lung function decline is often ascribed to remodeling of the airway, a more or less permanent change in the structure of the airway in response to an inflammatory stimulus (13). The sub-group of patients with Chronic Rhinosinusitis (14) with nasal polyps (CRSwNP) has elevated levels of IgE antibodies to *Staphylococcus aureus* enterotoxins (SAEs) (15, 16) and marked remodeling in the upper airway (17). Both chronic rhinosinusitis (18) and IgE antibodies to *Staph. aureus* enterotoxins (19) have been associated with higher risk of lung function decline and development of chronic obstructive pulmonary disease (COPD).

We have used post bronchodilator lung function data from the Global Allergy and Asthma European Network (GA^2^LEN) follow-up survey to explore the relation between decline in lung function and age, chronic sinusitis, asthma, smoking, and sensitization to common allergens.

## Materials and methods

### Study design and population

All participants included in this analysis participated in the GA^2^LEN clinical follow-up survey between September 2008 and April 2010. The GA^2^LEN follow-up study was a multi-center case–control study with participants selected from the respondents to an initial cross-sectional survey of a general population of adults aged 15–74 years living in 17 European cities and completed between June 2007 and May 2009 (20, 21).

### Sample selection

A random sample of eligible participants who indicated a willingness to be re-contacted was selected based on their responses to the initial postal survey (20, 21). The cases were participants who either had asthma, sinusitis or both and the controls were those who reported having neither.

All participants were aged 15–74 years and lived in a defined area. Participants with asthma had a history of either wheezing, shortness of breath or waking at night with breathlessness within the previous 12 months, and also reported a previous asthma diagnosis. Chronic rhinosinusitis cases had reported at least two of these symptoms: 1) nasal blockage, 2) nasal discharge, 3) facial pain or pressure, or 4) reduced sense of smell, at least one of which had to be either blockage or discharge and had reported having symptoms for at least for 12 weeks without remission (22). Controls lived in the same area, were aged 15–74 years but met neither of the criteria for asthma or rhinosinusitis. Each center was asked to recruit up to 120 cases each of patients with asthma only, CRS only, controls and 40 patients with both asthma and CRS based on the responses to the initial cross-sectional survey.

### Interviews and measurements

A self-administered questionnaire based on that of the European Community Respiratory Health Survey (ECRHS) (23), included questions on respiratory symptoms, family history of asthma, diagnosed asthma, use of asthma medications, history of allergy and sinusitis, smoking history and occupation. Height and weight were measured and skin tests for atopy were undertaken for D. Pteronyssinus, D. farinae, olive, artemisia (Stallergenes, Antony, France), birch, (Allergopharma, Reinbek, Germany) Blatella, (Leti, Madrid, Spain) Timothy grass, grass mix, cat, Alternaria, dog, Parietaria, and positive (Histamine) and negative (diluent) controls (ALK-Abelló, Hørsholm, Denmark). The tests were read at 15 min as the average of the longest diameter and that at right angles to it. All tests with an average diameter 3 mm greater than control were taken to be positive We categorized participants as atopic if they showed a positive test to any of the allergens.

### Lung function

Spirometry was carried out using the ndd EasyOne Spirometer (Ndd Laboratories, Zurich, Switzerland) with a daily calibration check. The FEV_1_, FVC, FEV_1_/FVC were measured. Volumes were measured at least three times each before and after administration of 200 micrograms of a short-acting bronchodilator (Salbutamol) and the two highest acceptable and reproducible maneuvers according to the ATS/ERS guidelines were analyzed (24).

### Quality control and training

A blow was accepted if the extrapolated volume was <5% of the FVC or 150 ml whichever was greater, and had a satisfactory pattern on manual review by an independent assessor. A blow with an acceptable pattern on inspection was one with no zero flow error, extra breaths or nonmaximal effort. In addition, an acceptable blow had to reach a plateau before termination and had to be free of excessive cough, especially in the first second. A blow was considered reproducible if the difference between the two highest FEV_1_ and FVC were each <150 ml.

Staff members from each center were trained centrally prior to involvement with the main data collection. Questionnaires were forward and back-translated, and the original questionnaires and the back-translations compared and reconciled.

### Data analysis

Analysis was undertaken using Stata version 11.0 (Stata Corp, College Station, TX, USA) (25). Those <20 years of age were excluded from the analysis. Cigarette smoking was quantified in pack years, defined as the number of cigarettes smoked per day divided by 20 and multiplied by the number of years that the participant smoked. Respondents who had never smoked cigarettes or had smoked <20 packs of cigarettes in their life time were regarded as ‘never smokers’, while others were categorized either as ‘former’ or ‘current’ smokers depending on their smoking status at the time of the survey.

Use of inhaled corticosteroids was classified as *regular* if a participant reported using them for at least two and half years in the last five years and *limited* if otherwise.

We regressed the postbronchodilator FEV_1_/FVC ratio against age, sex, height and case/control group. Interaction terms for age and case/control group were fitted, as were separate terms for patients with asthma who were or were not on regular inhaled corticosteroids. Inverse probability weights were used to adjust the results for the sampling design. After initial within-center analyses, the results were combined across centers using random effects meta-analysis. This provides an estimate of the consistency of the results between centers (*I*^2^) and provides more appropriate confidence intervals which take account of the clustering in the sample (26).

Local ethical committee approval was obtained in each center. Each participant was provided with an information sheet explaining the study and signed a consent form prior to taking part.

## Results

Table 1 gives the numbers in each of the case–control groups. In total 3337 eligible participants completed the clinical questionnaire and spirometry, of which 778 had asthma only, 399 had CRS only, 244 had both asthma and CRS and 1916 had neither CRS nor asthma.

**Table 1:** Recruitment of cases and controls by center

Center	Asthma	Chronic rhino-sinusitis	Asthma and chronic rhino-sinusitis	Controls	Total
Umea	117	41	29	168	355
Uppsala	114	42	32	202	390
Stockholm	101	26	34	232	393
Gothenburg	66	9	20	53	148
Helsinki	31	18	13	105	167
Odense	88	45	17	202	352
Lodz	23	27	5	111	166
Katowice	4	5	4	26	39
London	32	8	6	58	104
Southampton	32	3	5	33	73
Amsterdam	35	42	8	126	211
Ghent	17	28	13	88	146
Brandenburg	34	18	6	113	171
Duisburg	25	24	13	133	195
Skopje	5	14	3	95	117
Palermo	5	3	4	34	46
Coimbra	49	46	32	137	264
Total	778	399	244	1916	3337

Asthma cases had both a self-reported diagnosis of asthma and a history of either wheezing, shortness of breath or waking at night with breathlessness within the previous 12 months.

Chronic rhino-sinusitis cases were participants reporting at least two of these symptoms; 1) nasal blockage, 2) nasal discharge, 3) facial pain or pressure, or 4) reduced sense of smell, at least one of which had to be either blockage or discharge and symptoms had to have been present at least for 12 weeks without remission.

The controls lived in the same area but met neither of the criteria for diagnosis of asthma or sinusitis as above.

The mean age (SD) was 46.4 (14.5), 48.6(14.2), 47.4 (13.6), and 49.5 (14.6) for the groups with asthma only, CRS only, both asthma and CRS and those with neither asthma nor CRS respectively (Table 2). In all clinical categories, male smokers had exposure to more pack years of smoking than did female smokers. The proportion of respondents categorized as atopic were 73.1% (asthma only), 45.6% (CRS only), 64.1% (both asthma and CRS) and 44.7% (neither asthma nor CRS).

**Table 2:** Characteristics of cases and controls

Variable	Asthma	Chronic rhino-sinusitis	Asthma and chronic rhino-sinusitis	Controls
Age (mean; SD)	46.37 (14.50)	48.60 (14.22)	47.44 (13.58)	49.48 (14.61)
Sex (% male)	40.80	46.60	37.45	44.93
Height (mean; SD)	170.12 (9.64)	170.37 (10.33)	168.56 (10.33)	170.23 (9.65)
Smoking (Men)				
Never (%)	44.48	42.70	42.86	43.29
Former (%)	41.01	35.14	45.05	34.42
Current (%)	14.51	22.16	12.09	22.29
Smoking (Women)				
Never (%)	53.81	49.53	50.00	56.33
Former (%)	30.72	25.94	28.29	28.35
Current (%)	15.47	24.53	21.71	15.32
Pack years in ever smokers (men)	10.82	14.41	15.58	12.96
Pack years in ever smokers (women)	6.88	9.75	9.76	7.03
Atopy (%)	73.11	45.68	64.16	44.73
Atopy (%) aged<40	84.48	55.05	82.05	56.16
Atopy (%) aged ≥ 40	66.08	41.54	54.42	39.77
Nasal discharge (%)	5.02	54.39	65.16	4.13
Blocked nose (%)	15.94	90.73	92.62	11.61
Nasal discharge or blocked nose (%)	20.98	100	100	15.74
Facial pain/pressure or reduced sense of smell (%)	9.41	86.96	79.92	9.67
Steroid use				
None/limited (%)	65.59	96.73	60.53	96.31
Regular (%)	34.37	3.32	39.47	3.73
Postbronchodilator FEV_1_ (Men) L	3.53	3.80	3.36	3.69
Postbronchodilator FEV_1_ (Men)% predicted	88.23	95.72	84.23	95.38
Postbronchodilator FVC (Men) L	4.79	4.84	4.53	4.74
Postbronchodilator FVC (Men)% predicted	94.42	96.06	89.78	95.09
Postbronchodilator FEV_1_/FVC (Men)%	73.66	78.13	73.01	77.71
Postbronchodilator FEV_1_/FVC (Men)% of% predicted	93.96	99.91	93.16	100.08
Postbronchodilator FEV_1_ (Women) L	2.73	2.76	2.67	2.82
Postbronchodilator FEV_1_ (Women)% predicted	91.10	96.66	91.58	97.14
Postbronchodilator FVC (Women) L	3.52	3.48	3.37	3.52
Postbronchodilator FVC (Women)% predicted	95.42	97.82	93.64	97.28
Postbronchodilator FEV_1_/FVC (Women)%	77.19	79.19	78.76	80.33
Postbronchodilator FEV_1_/FVC (Women)% of% predicted	94.80	98.42	97.11	99.53

Asthma cases had both a self-reported diagnosis of asthma and a history of either wheezing, shortness of breath or waking at night with breathlessness within the previous 12 months.

Chronic rhino-sinusitis cases were participants reporting at least two of these symptoms; 1) nasal blockage, 2) nasal discharge, 3) facial pain or pressure, or 4) reduced sense of smell, at least one of which had to be either blockage or discharge and symptoms had to have been present at least for 12 weeks without remission.

The controls lived in the same area but met neither of the criteria for diagnosis of asthma or sinusitis as above.

Use of inhaled corticosteroids was classified as *regular* if a participant reported using it for at least two and half years in the last five years and *limited* if otherwise. FEV_1_: forced expiratory volume in 1 s; FVC: forced vital capacity; Atopy was considered positive if ≥3 mm reaction size to any of the allergens.

FEV_1_ and FVC percent predicted are based on the NHANES prediction equations.

Table 3 shows the cross-sectional association of postbronchodilator FEV_1_/FVC ratio with age, sex, height, atopy and group. There is a significant decrease of 0.8% per 10 pack years smoked (*P* < 0.001), and current smokers have an additional 2% lower FEV_1_/FVC ratio compared with never smokers. The decrement in the ratio for those with asthma is much larger at, −4.1% *P* < 0.001, whether or not they also have chronic rhinosinusitis. There are smaller nonsignificant reductions in those with chronic rhinosinusitis (−0.6%) and atopy (−0.04%).

**Table 3:** Meta-analysis of postbronchodilator FEV_1_/FVC ratio against age, sex, height, diagnosis and smoking history in 3337 study participants

Variables	Estimates	Confidence limits (95%)		*P* value*	*I*^2^	*P* value (heterogeneity)†
Age/year from 20 (years)	−0.254	−0.286	−0.221	<0.001	42.8	0.036
Sex						
Female	–	–	–	–	–	–
Male	0.119	−0.752	0.990	0.789	0.0	0.595
Height/m from 170 cm	−0.087	−0.130	−0.044	<0.001	0.0	0.520
Cases grouping						
Controls (ref)	–	–	–	–	–	–
Asthmatic	−4.090	−5.025	−3.155	<0.001	16.7	0.266
Sinusitis	−0.592	−1.487	0.303	0.195	0.0	0.842
Both Asthma & Sinusitis	−2.773	−3.812	−1.734	<0.001	0.0	0.593
Smoking (per pack year)	−0.084	−0.124	−0.043	<0.001	53.7	0.006
Smoking status						
Never (ref)	–	–	–	–	–	–
Former	−0.732	−2.186	0.723	0.324	68.9	<0.001
Current	−1.991	−3.032	−0.950	<0.001	2.7	0.421
Atopy	−0.044	−0.728	0.640	0.899	15.6	0.283

FEV_1_, forced expiratory volume in 1 s; FVC, forced vital capacity.

* Effect Estimate.

† Heterogeneity of effects between centers.

When the interactive effects of age are taken into account (Table 4), neither the main effect of atopy nor the interactive effects of age with atopy were significant (model 1), and the main effect for atopy indicated a slightly higher FEV_1_/FVC ratio 1.00% (95% CI: −0.33, 2.32). However, model 2 (Table 4) also shows a significant interaction between age and asthma, accounting for an estimated −0.135% lower FEV_1_/FVC ratio for each year of age, which is equivalent to the estimated effect of smoking between one and two packs of cigarettes per day. This is so whether or not they have concurrent chronic rhinosinusitis.

**Table 4:** Meta-analysis of postbronchodilator FEV_1_/FVC ratio including interactions for age and atopy and age and diagnosis

Variables	Model 1						Model 2					
	Estimates	Confidence limits (95%)		*P* value*	*I*^2^	*P* value (heterogeneity)†	Estimates	Confidence limits (95%)		*P* value*	*I*^2^	*P* value (heterogeneity)†
Age/year from 20 (years)	−0.250	−0.291	−0.209	<0.001	37.8	0.058	−0.241	−0.274	−0.207	<0.001	39.3	0.049
Sex												
Female	–	–	–	–	–	–	–	–	–	–	–	–
Male	0.062	−0.806	0.929	0.889	4.2	0.405	−0.192	−1.024	0.640	0.651	0.0	0.721
Height/m	−0.097	−0.145	−0.050	<0.001	15.4	0.273	−0.088	−0.134	−0.042	<0.001	12.5	0.307
Cases grouping												
Controls (ref)	–	–	–	–	–	–	–	–	–	–	–	–
Asthmatic	−4.001	−5.018	−2.984	<0.001	27.3	0.143	−0.128	−1.508	1.252	0.856	0.0	0.948
Sinusitis	−0.307	−1.229	0.614	0.513	4.2	0.406	−1.231	−3.980	1.518	0.380	55.4	0.004
Both Asthma & Sinusitis	−2.942	−4.070	−1.815	<0.001	10.1	0.335	−3.413	−9.937	3.111	0.305	87.4	<0.001
Age*case interaction												
Controls (ref)							–	–	–	–	–	–
Asthmatic*Age							−0.135	−0.186	−0.084	<0.001	0.0	0.658
Sinusitis*Age							0.030	−0.063	0.122	0.529	52.8	0.006
Both Asthma & Sinusitis*Age							−0.003	−0.233	0.228	0.982	85.5	<0.001
Smoking (per pack year)	−0.086	−0.123	−0.049	<0.001	50.5	0.009	−0.084	−0.120	−0.048	<0.001	42.9	0.031
Smoking status												
Never (ref)	–	–	–	–	–	–	–	–	–	–	–	–
Former	−0.439	−1.710	0.832	0.498	60.0	0.001	−0.457	−1.731	0.818	0.482	59.8	0.001
Current	−1.971	−2.971	−0.970	<0.001	0.0	0.518	−2.235	−3.238	−1.231	<0.001	0.4	0.449
Atopy												
No Atopy (ref)	–	–	–	–	–	–						
Atopy	0.996	−0.326	2.318	0.140	6.1	0.385						
Atopy*Age	−0.034	−0.083	0.014	0.166	17.8	0.260						

Atopy was considered positive if ≥3 mm reaction size to any of the allergens.

FEV_1_, forced expiratory volume in 1 s; FVC, forced vital capacity.

* Effect Estimate.

† Heterogeneity of effects between centers.

Table 5 shows the effects of age in those with asthma divided into those who were taking regular steroids and those who were not. Those who were on regular steroids had a steeper decline in FEV_1_/FVC with age; (−0.28% per annum, 95% CI −0.08, −0.49) compared with those on limited/no corticosteroid (−0.15% per annum, 95% CI −0.03, −0.27), though there is considerable overlap in the estimates and heterogeneity between centers is substantial.

**Table 5:** Meta-analysis of postbronchodilator FEV_1_/FVC ratio including interaction for age, diagnosis and steroid use

Variables	Model 3					
	Estimates	Confidence limits (95%)		*P* value*	*I*^2^	*P* value (heterogeneity)†
Age/year from 20(years)	−0.239	−0.272	−0.206	<0.001	36.4	0.067
Sex						
Female	–	–	–	–	–	–
Male	−0.127	−0.961	0.707	0.765	0.0	0.659
Height/m	−0.091	−0.135	−0.046	<0.001	8.4	0.356
Cases grouping						
Controls (ref)	–	–	–	–	–	–
Asthmatic (limited/no steroid use)	−0.281	−1.753	1.191	0.708	0.0	0.930
Asthmatic (regular steroid use)	−0.048	−3.476	3.380	0.978	56.5	0.004
Sinusitis	−1.180	−3.886	1.526	0.393	54.1	0.005
Both Asthma & Sinusitis	−3.622	−10.181	2.936	0.279	87.3	<0.001
Age*case interaction						
Controls (ref)	–	–	–	–	–	–
Asthmatic (limited/no steroid use) *Age	−0.150	−0.267	−0.033	0.012	75.4	<0.001
Asthmatic (regular steroid use) *Age	−0.281	−0.485	−0.076	0.007	85.5	<0.001
Sinusitis*Age	0.028	−0.062	0.117	0.545	50.5	0.009
Both Asthma & Sinusitis*Age	0.006	−0.224	0.237	0.958	85.3	<0.001
Smoking (per pack year)	−0.084	−0.120	−0.049	<0.001	39.0	0.051
Smoking status						
Never (ref)	–	–	–	–	–	–
Former	−0.479	−1.716	0.759	0.449	56.9	0.002
Current	−2.261	−3.269	−1.253	<0.001	0.0	0.629

Regular steroid users were those who reported use of steroid inhalers for at least two and half years over the last five years.

FEV_1_, forced expiratory volume in 1 s; FVC, forced vital capacity.

* Effect Estimate.

† Heterogeneity of effects between centers.

Table 6 shows the effect of atopy and steroid use on FEV_1_/FVC ratio. Among asthmatic participants with limited or no steroid use, the nonatopic group had a steeper decline of FEV_1_/FVC ratio with age compared with the atopic group (−0.31% *vs* −0.06%; *P* < 0.05). A similar trend was also noted in those on regular steroid, though it did not attain statistical significance.

**Table 6:** Meta-analysis of postbronchodilator FEV_1_/FVC ratio including interaction for age, diagnosis, atopy and steroid use

Variables	Model 4					
	Estimates	Confidence limits (95%)		*P* value (estimate)*	*I*^2^	*P* value (heterogeneity)†
Age/year from 20 years	−0.237	−0.270	−0.205	<0.001	34.1	0.083
Sex						
Female	–	–	–	–	–	–
Male	−0.120	−0.963	0.723	0.780	0.0	0.724
Height/m	−0.090	−0.136	−0.044	<0.001	11.1	0.325
Cases grouping						
Controls (ref)	–	–	–	–	–	–
Asthmatic (no atopy; limited/no steroid use)	3.227	−4.408	10.862	0.408	91.0	<0.001
Asthmatic (atopic; limited/no steroid use)	−0.645	−2.164	0.873	0.405	0.0	0.865
Asthmatic (no atopy; regular steroid use)	1.628	−6.628	9.883	0.699	79.2	<0.001
Asthmatic (atopic; regular steroid use)	−1.044	−4.561	2.473	0.561	67.9	<0.001
Sinusitis	−1.130	−3.796	1.537	0.406	51.7	0.009
Both Asthma & Sinusitis	−3.670	−10.319	2.978	0.279	87.3	<0.001
Age*case interaction						
Controls (ref)	–	–	–	–	–	–
Asthmatic (no atopy; limited/no steroid use) *Age	−0.306	−0.548	−0.065	0.013	92.5	<0.001
Asthmatic(atopic; limited/no steroid use)*Age	−0.060	−0.114	−0.006	0.029	0.0	0.491
Asthmatic (no atopy; regular steroid use)*Age	−0.395	−0.835	0.045	0.078	98.4	<0.001
Asthmatic (atopic; regular steroid use)*Age	−0.243	−0.495	0.008	0.058	92.9	<0.001
Sinusitis *Age	0.026	−0.063	0.115	0.564	48.7	0.013
Both Asthma & Sinusitis*Age	0.007	−0.226	0.241	0.950	85.3	<0.001
Smoking (per pack year)	−0.085	−0.120	−0.050	<0.001	40.8	0.041
Smoking status						
Never (ref)	–	–	–	–	–	–
Former	−0.429	−1.660	0.802	0.495	55.4	0.003
Current	−2.142	−3.154	−1.130	<0.001	0.0	0.621

Atopy was considered positive if ≥3 mm reaction size to any of the allergens.

FEV_1_, forced expiratory volume in 1 s; FVC, forced vital capacity.

* Effect Estimate.

† Heterogeneity of effects between centers.

## Discussion

**Figure 1:**
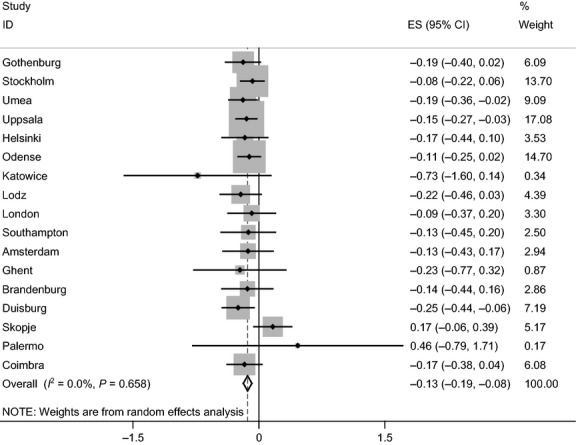
Forest plot showing meta-analysis of postbronchodilator FEV_1_/FVC ratio for the interaction between age and asthma.

**Figure 2:**
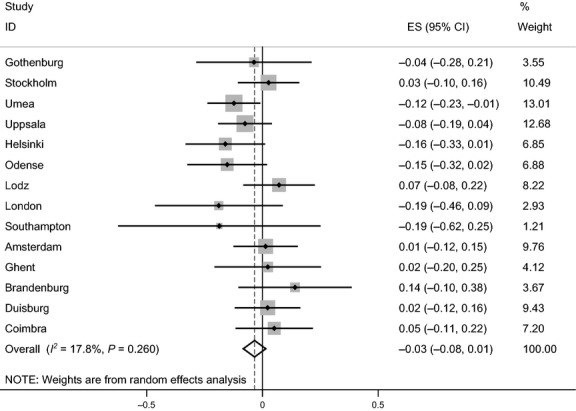
Forest plot showing meta-analysis of postbronchodilator FEV_1_/FVC ratio for the interaction of age and atopy.

We have shown from this multicenter study of adults living in Europe that participants with asthma have a lower postbronchodilator FEV_1_/FVC ratio than controls and this difference increased with age (Fig. 1). The size of the change with age was comparable to that among those smoking 20 cigarettes per day. Participants who were atopic did not have lower lung function when adjusted for age and sex (Fig. 2), nor did those with chronic rhinosinusitis.

This is a large study of postbronchodilator spirometry in a widely representative European population spread across many centers. The lung function data itself was quality assured, all tests being checked by an independent reader for compliance with ATS/ERS standards (24). Skin prick tests were standardized with common training and common allergen solutions.

In cross-sectional studies, the interpretation of associations with age is not straightforward. In the simple case of lung function, this can mean a decline of lung function with age, better survival rate among those with poor lung function, an unlikely explanation, or higher age-adjusted lung function in those who were born most recently, a so-called ‘birth cohort’ effect. The analyses presented here are more complicated in that they represent a difference in association with age between two or more risk groups. So the relatively faster decline with age among the smokers could be due to an accelerated decline with age among smokers, better survival in smokers with a low lung function, another unlikely explanation, or a relatively better survival of smokers compared with nonsmokers among those born later. This last explanation might be plausible if smoking habits had become less harmful in later generations, though the large size of this effect and what is already known about the effects of smoking also make this explanation unlikely. The cases for asthma and atopy are less certain because there is less evidence from other studies. However, the straightforward interpretation of both findings as effects associated with ageing fits earlier reports from both cross-sectional and longitudinal studies and helps to reconcile their findings. Interpretation of the findings on corticosteroid use is less clear because of the less precise nature of the questions and the problem of confounding by indication. These results help to reconcile the results of Burrows no increased decline in those with atopy with the results of others who have found a rapid decline of postbronchodilator lung function in those with asthma (1, 27). Like Burrows we did not find any association between atopy and lower lung function with age. If anything, the atopic group had better lung function than expected.

Shirtcliffe et al. (12) reported similar results from New Zealand though they did not look at the interaction with age. Like that study, which was also a study of postbronchodilator lung function in a general population of adults under the age of 75 years, we found an effect of asthma at least equivalent to that of a moderate smoker, but found no deficit in lung function attributable to atopic sensitization.

Unlike Lange and de Marco (8, 9) we did not find lower decline in lung function with age among asthmatic participants taking regular inhaled corticosteroids. An association between ICS use and severity is common in observational studies of asthma and is generally interpreted as evidence that those with the most severe disease are more likely to be prescribed and to take these medications. Both the papers by Lange et al. and de Marco et al. have the advantage of being true longitudinal studies, but they used prebronchodilator measures of lung function and this may have influenced their results, particularly if there was a greater likelihood of using these medications at the end of the period of observation than at the beginning.

We did not find a lower lung function with age in those with CRS. Loss of lung function with age in asthma is often ascribed to remodeling of the airway. In CRS, there is prominent remodeling of the upper airway and there has been some evidence that those with the condition do have lower ventilatory function. Our results do not support this finding. However, the loss of lung function previously noted was among the sub-group of patients with nasal polyps (28, 29), and we are unable to distinguish clearly between these sub-groups in this study.

Though some studies report to the contrary (30), it is of interest that the effect associated with ‘asthma and sinusitis’ appears to be less than the effect of asthma alone, and this might suggest a protective effect of sinusitis. Such a protective effect is not seen, however, for sinusitis alone and the difference is also less marked, and not significant, when we consider the interaction effects with age.

Drawing generalizable conclusions from large multicenter studies depends on consistent associations being found in all centers. The associations between the postbronchodilator FEV_1_/FVC and current smoking, asthma and atopy and the interactions of both asthma and atopy with age in models 1, 2, and 3 are consistent across the sites. The associations with sinusitis, with pack years smoking and the interactions with corticosteroid use are not consistent. This may be due to real differences in the exposures between sites, for instance the type of tobacco smoked or patterns of corticosteroid use, or it could be due to varying levels of uncontrolled confounding in some centers. Lack of consistency in these analyses leads us to put less emphasis on these conclusions than on the principal conclusions. We did not perform CT scans of sinuses or endoscopy on the cases with CRS and cannot exclude the chance of misclassification in some cases, however, the criteria applied in this study have been used in previous studies and have been shown to be sensitivity for CRS (20, 22).

In conclusion, we have shown that an age-related reduction in postbronchodilator lung function is associated with asthma as defined in this study, but not with atopy. There is no reason to expect a greater than normal decline in lung function in atopic patients who do not have asthma; the extent to which an accelerated decline in lung function can be prevented by the use of inhaled steroids has yet to be clearly established. Prospective controlled studies in asthma would be required to evaluate fully the impact of atopy and therapeutic intervention on this change in lung function.
